# Down-regulation of immune checkpoints by doxorubicin and carboplatin-containing neoadjuvant regimens in a murine breast cancer model 

**DOI:** 10.22038/ijbms.2021.54383.12221

**Published:** 2021-04

**Authors:** Sanambar Sadighi, Ramezanali Sharifian, Monireh Kazemimanesh, Ahad Muhammadnejad, Zahra Shahosseini, Saeid Amanpour, Samad Muhammadnejad

**Affiliations:** 1 Department of Medical Oncology, Cancer Research Center, Cancer Institute of Iran, Tehran University of Medical Sciences, Tehran, Iran; 2 Department of Hematology and Oncology, Vali-e-Asr Hospital, Tehran University of Medical Sciences, Tehran, Iran; 3 Department of Molecular Virology, Pasteur Institute of Iran, Tehran, Iran and Université Toulouse III Paul Sabatier, INSERM U1037, Cancer Research Centre of Toulouse (CRCT), Toulouse, France; 4 Cancer Biology Research Center, Cancer Institute of Iran, Tehran University of Medical Sciences; 5 Department of Medical Biotechnology, School of Allied Medical Sciences, Iran University of Medical Sciences, Tehran, Iran; 6 Gene Therapy Research Center, Digestive Diseases Research Institute, Tehran University of Medical Sciences, Tehran, Iran; 7 Pediatric Cell Therapy Research Center, Tehran University of Medical Sciences, Tehran, Iran

**Keywords:** Animal model, Breast neoplasms, Immune checkpoints, Neoadjuvant chemotherapy, Tumor-infiltrating - lymphocytes

## Abstract

**Objective(s):**

Immune checkpoint expression on tumor-infiltrating lymphocytes (TILs) has a correlation with the outcome of neoadjuvant chemotherapy (NAC) in breast cancer. However, the reciprocal effect of these regimens on the quality and quantity of immune checkpoints has hitherto not been addressed. We aimed to evaluate the impact of three NAC regimens on TILs and immune checkpoints in a murine triple-negative breast cancer model.

**Materials and Methods:**

Syngeneic model of locally-advanced breast cancer was established in immunocompetent mice using a 4T1 cell line. Tumor-bearing animals were treated with human-equivalent dosages of doxorubicin, paclitaxel, paclitaxel and carboplatin combination, and placebo. Infiltration of CD3^+^, CD8^+^, and FoxP3^+^ cells into the tumor was assessed by immunohistochemistry. Expression of immune checkpoints, including *PD-1*, *CTLA-4,* and *TIM-3*, was evaluated by real-time PCR.

**Results:**

Doxorubicin led to a significant (*P*<0.01) increase in the percentage of the stromal infiltrating CD3^+^ and CD8^+^ lymphocytes. Doxorubicin also suppressed significantly (*P*<0.05) the relative expression of *PD-1* compared with the placebo. *PD-1 *expression was significantly (*P*<0.05) lower in the group treated with paclitaxel and carboplatin combination as compared with the placebo. The relative expression of *TIM-3 *was significantly (*P*<0.05) suppressed in doxorubicin-treated mice in comparison with other interventions.

**Conclusion:**

Our findings hypothesize that NAC with doxorubicin may potentiate antitumor immunity not merely by recruitment of TILs, but via down-regulation of PD-1 and TIM-3 checkpoints. Carboplatin-containing NAC may suppress *PD-1* as well.

## Introduction

Neoadjuvant chemotherapy (NAC) has been playing an essential role in the treatment of breast cancer (BC) throughout the last decade. The main objective of this approach is to downstage BCs, permitting a less-invasive surgical intervention. It might also improve survival outcomes via elimination of distant occult micrometastases (1). The mainstay of present NAC treatment includes anthracyclines and taxanes administered either successively or simultaneously for up to 8 cycles (2). Recent studies have suggested that not only do these chemotherapeutic regimens exert their clinical response by direct cytotoxic effects on neoplastic cells, but also do so via augmentation of antitumor immunity in the microenvironment of malignancy (3). 

There is compelling evidence to support the crucial role of the immune tumor microenvironment in the initiation and progression of cancer. Some breast tumors reportedly have remarkable tumor-infiltrating lymphocytes (TILs) (4). Infiltrating lymphocytes entail different cellular subsets such as CD8+ cytotoxic T cells and CD4^+^ lymphocytes, including regulatory T (T_Reg_) cells. CD8^+^ T cells are assumed to trigger apoptosis in malignant cells via secretion of perforin and granzyme B. In clinical studies higher intratumoral CD8^+^ T cell numbers correlated with favorable prognosis (5). T_Reg_ lymphocytes play a part in immunoediting by inhibiting immune stimulation and specific immune reactions. This cellular subset of lymphocytes has been further characterized as FoxP3^+^ T cells (6). The prognostic significance of FoxP3^+^ T cells remains highly controversial (7). However, the ratio of CD8+ T lymphocytes to T_Reg_ cells appears to be associated with a favorable prognosis in the majority of cancers (5). The ratio of different components of antitumor immunity is somewhat governed by immune checkpoints such as programmed death receptor-1 (PD-1), cytotoxic T lymphocyte-associated protein-4 (CTLA-4), and T cell Ig and mucin domain-3 (TIM-3). These co-inhibitory receptors have the primary function in the modulation of immune activation. Immune checkpoints often suppress antitumor immunity. Therefore, inhibition of these receptors has led to a significant breakthrough in the treatment of cancer (8, 9). 

Among different molecular subtypes of BC, the triple-negative one is most likely to have predominant lymphocyte infiltration (10). TIL counts reportedly predict the outcome of NAC in triple-negative BC (11). In addition, inhibition of immune checkpoints has shown impressive antitumor activity and durable long-term disease control in this molecular subtype of BC (12). 

**Figure 1:**
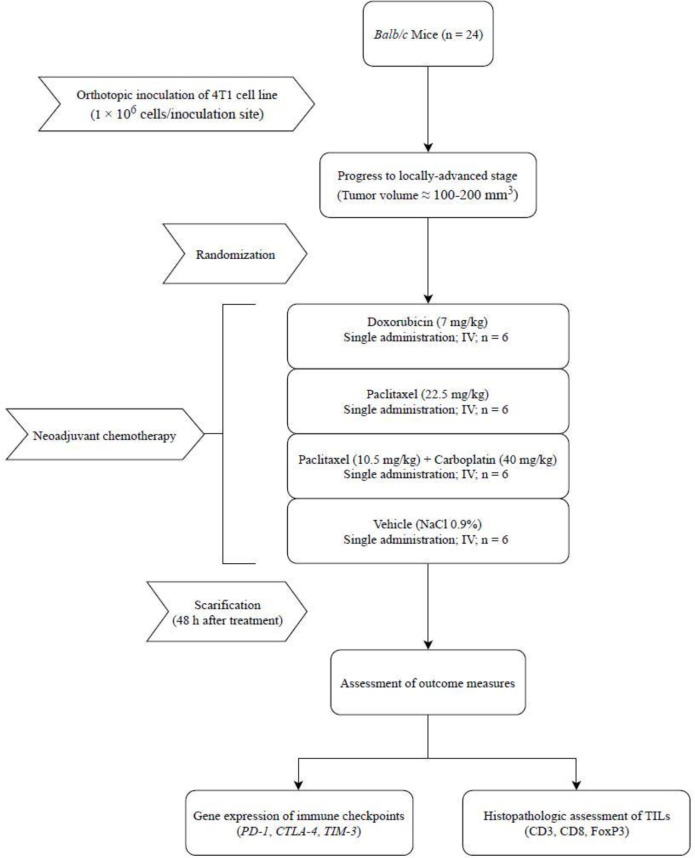
Experiment protocol diagram. In this experiment, the effects of three neoadjuvant chemotherapy regimens on the infiltration of lymphocytes and relative expression of immune checkpoints were assessed in the syngeneic model of 4T1 mammary gland tumor

Although the prognostic role of TILs and immune checkpoints in the clinical response to NAC regimens in triple-negative BC has been touched in different works (13, 14), the reciprocal effects of these regimens on TILs and immune checkpoints have not been addressed. Therefore, we intended to evaluate the influence of three NAC regimens including doxorubicin, paclitaxel, and carboplatin-paclitaxel combination on TILs and immune checkpoints in a syngeneic mouse model of triple-negative mammary gland tumor. In this study, doxorubicin and paclitaxel were used as backbone drugs in the neoadjuvant treatment of BC. Although carboplatin is not a standard agent in the mentioned setting (15), we added carboplatin to paclitaxel to induce chromosomal breakage in the triple-negative specimen. The experimental design has been shown in Figure 1**.**

## Materials and Methods

***Mice***

Six- to eight-week-old, female BALB/c mice were purchased from the Pasteur Institute of Iran. The animals were kept in the temperature-controlled animal facility of the Cancer Institute of Iran on a 12-hr photoperiod. The animals were fed *ad libitum*. All processes accomplished in this experiment involving animals were under the ethical standards of the Ethical Committee of Tehran University of Medical Sciences clearance (code: 184060).

***Cell preparation and tumor establishment***

4T1 mouse mammary carcinoma cell line, which is equivalent to human triple-negative BC (16), was purchased from the National Cell Bank of Iran (Pasteur Institute of Iran). Authentication of the cell line was performed using short tandem repeat analysis in the cell bank. The cells were grown in DMEM medium supplemented with 10% fetal calf serum (both from Gibco, USA). We inoculated twenty-four mice in the mammary fat pad (left *regio mammaria abdominalis*) with 1 × 10^6^ early passage 4T1 cells collected from culture flasks by treatment with 0.25% trypsin and one mM EDTA (Gibco, USA). Morphometric growth of tumor was measured by digital calipers, and tumor volumes were determined by the formula *V* (mm^3^) = *L *(length) × *W*^2^ (width) × 0.52 (17)

***Neoadjuvant chemotherapy***

When tumor volumes became 100–200 mm^3^, the animals were randomly assigned to one of four groups: doxorubicin, paclitaxel, paclitaxel + carboplatin (PCb), and placebo (6 mice per group). Sample size calculation was based on the resource equation method (18). We carried out randomization by Research Randomizer (available at http://www.randomizer.org). Regarding local invasion presented in our pilot studies between 100–200 mm^3^, we considered this range of tumor volume for the 4T1 syngeneic model as equal to locally advanced BC in humans, the stage which has been adopted for neoadjuvant therapy. Dose back-translation of neoadjuvant regimens from human to mouse was done using the following formula:

Indeed, the K_m_ constant is the proportion of body weight (kg) to body surface area (m^2^) in each species. K_m_ values for human and mouse were considered as 37 and 3, respectively (19). The doxorubicin-treated group received DOXO-cell^®^ (Germany) at a dosage of 7 mg/kg. Paclitaxel (Paclitaxel Stragen^®^; Sobhan Oncology, Iran) was administered at doses of 22.5 mg/kg and 10.35 mg/kg as monotherapy or in combination with carboplatin, respectively. The dosage of carboplatin (MYLAN, France) was determined as 40 mg/kg. Physiological saline solution was used as a placebo. All drugs were administered intravenously via tail vein at a volume of 100 µl. To ensure that the person administering these medications did not know what the syringes contain, the drugs were prepared and labeled by an independent investigator. Mice were sacrificed using carbon dioxide asphyxiation 48 hr after treatment, and tumors were harvested. The reason for assessment of short-term effects of NAC regimens on outcome measures was to minimize the long-term lymphotoxic effects of these drugs on cellular components of antitumor immunity. According to our pilot work, this post-treatment time was enough to see the alterations of antitumor immunity. Each sample was divided into two parts; one part was assigned to histopathological study, and the other one was devoted to gene expression assessment.

***Histopathology***

That part of tumor specimen devoted to histopathological study was instantly fixed with 4% formaldehyde in 0.1 M phosphate-buffered saline. After preparation of formalin-fixed paraffin-embedded blocks, the specimens were stained using Hematoxylin and Eosin (H&E). The presence of TILs was assessed in these slides. 

To prepare immunohistochemical sections, paraffin-embedded blocks were chilled in an ice-water mixture for half an hour. Then, 4 µM sections were made. Subsequently, the sections were mounted on slides and fixed by heat at 37 °C. Following deparaffinization and rehydration of the specimens, immunohistochemical staining was performed according to instructions of the kit’s manufacturer (Biorbyt, United Kingdom) by CD3 (orb10313), CD8 (orb10325), and FoxP3 (orb34127). The different immunophenotypes of % stromal TILs were determined by a pathologist in a blinded manner as previously described by Vittoria Dieci *et al.* (20). Regarding the clinical importance of intratumoral CD8 to CD3 and FoxP3 to CD3 fractions, we also calculated these ratios.

***Gene expression***

The other part of tumor tissue specimens devoted to gene expression analysis were fixed in RNAlater® RNA stabilization reagent (QIAGEN, Germany) and kept at -20 °C up to the extraction of RNA. 

Total RNA extraction was performed by trizol, in accordance with the manufacturer’s protocol (Invitrogen, USA). The RNA quality and concentrations were determined using a Picodrop spectrophotometer (Ltd Cambridge, UK), and contaminating DNA was removed using Rnase-free DNase I (SINACLON, Iran). Similar amounts of RNA (1 μg) were used for cDNA synthesis. cDNA was synthesized from mRNA in 20 μl reaction volumes with random priming (in the same conditions during one day) by RevertAid first-strand cDNA synthesis kit (Thermo Scientific, USA).

To determine mRNA expression of *PD-1*, *TIM-3*, *CTLA-4,* and *β-actin* (as an internal control), we used the sets of primers whose sequences are presented in Table 1. Real-time quantitative PCR was carried out in an Applied Biosystems 7500 Fast, with a 20 μl reaction mixture containing 1 μl cDNA of each sample, SYBR Green master mix 1X (Takara, Japan), and 2 pM of each primer, under the following circumstances: 95 °C for 30 sec succeeded by 40 cycles of 95 °C for 5 sec , 60 °C for 34 sec, and a melting curve collection. Each experiment was performed in duplicate. The cycle number crossing the reporter fluorescence cycle threshold (CT) was considered for quantitative measurement. We repeated the experiments of samples whose CT values of *β-actin* were more than 28 or differences of CT values of duplicate in each gene were more than 1. 

The relative expression of the above-mentioned immune checkpoints was determined after normalizing to the expression of *β-actin* at the same samples using the 2^-ΔΔCT^ relative quantification method (21).

***Statistical analyses***

Normality tests of data were assessed by a two-tailed D’Agostino and Pearson omnibus K2 test. We analyzed normally distributed data with one way ANOVA followed by Tukey’s multiple comparisons test. Where the normal distribution test failed, the Kruskal-Wallis test was applied followed by Dunn’s multiple comparisons test. All *P*-values of less than 0.05 were assumed statistically significant. The statistical analyses were accomplished using GraphPad Prism 6 software.

## Results

***Histopathology***

**Figure 2:**
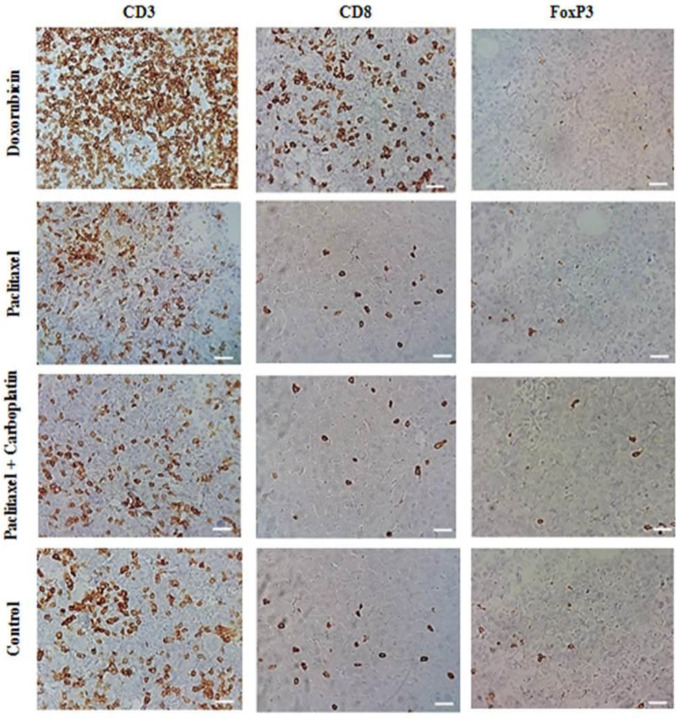
Immunohistochemistry micrographs of infiltrating CD3^+^, CD8^+^, and FoxP3^+^ lymphocytes into 4T1 mammary gland tumor after neoadjuvant chemotherapy. Dark-brown cells indicate immunoreactive lymphocytes. Increased infiltration of CD3^+^ and CD8^+^ cells into tumor tissue is evident in the doxorubicin-treated group. Scale bar, 20 µM

**Figure 3:**
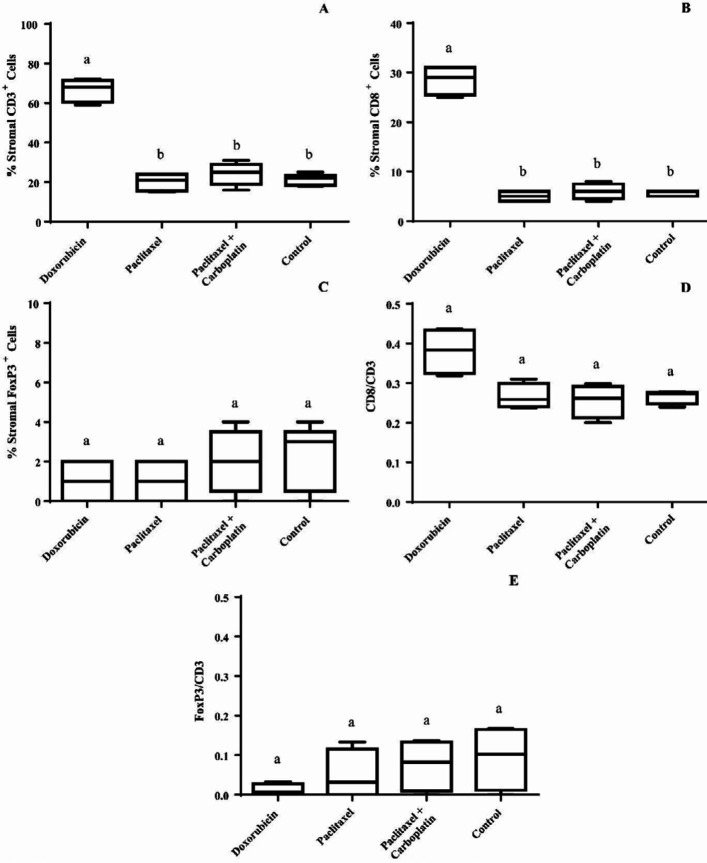
Box plot of percent stromal infiltrating lymphocytic subpopulations and their ratios in 4T1 mammary gland tumor after neoadjuvant chemotherapy. Tumor specimens were stained for CD3, CD8, and FoxP3 markers and quantified in each slide as stromal cell percentage. (A) Percent stromal CD3^+^ lymphocytes. (B) Percent stromal CD8^+^ Lymphocytes. (C) Percent stromal FoxP3^+^ lymphocytes. (D) CD8 to CD3 ratio. (E) FoxP3 to CD3 ratio. Different lower-case letters denote significant differences by Dunn’s multiple comparison test for the evaluated groups (*P*<0.01)

Immunohistochemistry (IHC) micrographs of infiltration of CD3^+^, CD8^+^, and FoxP3^+^ cells into the mouse mammary gland tumors are represented in Figure 2. Doxorubicin led to a significant increase (*P*<0.01) in the percentage of stromal infiltrating CD3^+^ lymphocytes compared with the other interventions (Figure 3A). The other treatments did not cause any significant alterations in the infiltration percent of this cell subpopulation.

Doxorubicin significantly (*P*<0.01) increased the percentage of stromal infiltrating CD8^+^ lymphocytes compared with other interventions (Figure 3B). Other chemotherapeutic regimens did not show any statistically meaningful change in the absolute count of CD8^+^ cells.

The population of FoxP3 cells did not show any statistically significant change after treatment of tumor-bearing mice with different chemotherapeutic regimens (Figure 3C). Although doxorubicin increased CD8^+ ^/ CD3^+^ ratio and decreased FoxP3^+^/ CD3^+^ proportion, neither doxorubicin nor other interventions showed any statistically significant alteration in these ratios (Figuresf 3D-E).

***Gene expression***

**Figure 4:**
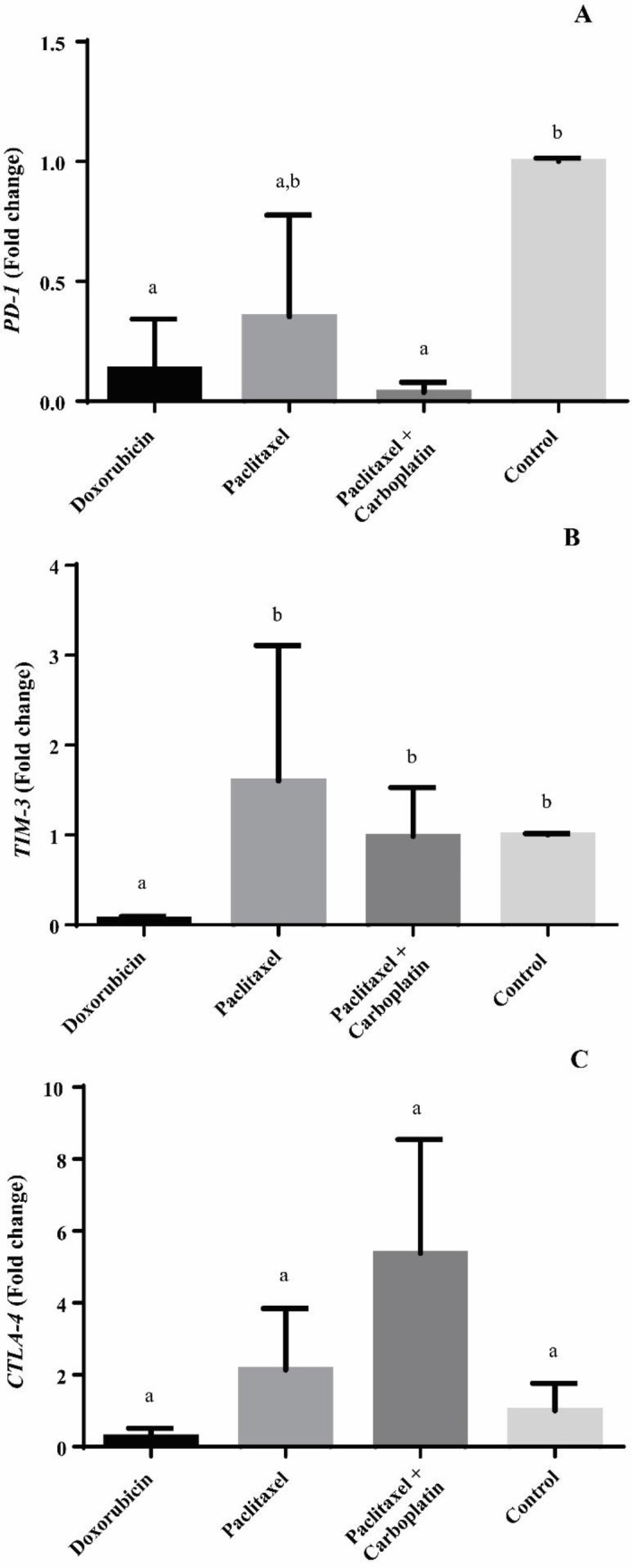
Means (± SEM) of the relative (fold change) gene expression in 4T1 mammary gland tumor after neoadjuvant chemotherapy. (A) PD-1 relative expression. (B) TIM-3 relative expression. (C) CTLA-4 relative expression. (a-b) Different lower-case letters denote significant differences by Tukey’s test for the evaluated groups (*P*<0.05)

Chemotherapy with doxorubicin led to a significant (*P*<0.05) decrease in the relative expression of *PD-1* in tumor tissues in contrast to the vehicle (Figure 4A). *PD-1* expression was significantly (*P*<0.05) lower in the group treated with paclitaxel and carboplatin combination as compared with the placebo (Figure 3A). However, monotherapy with paclitaxel did not influence the relative expression of this immune checkpoint.

Doxorubicin significantly (*P*<0.05) reduced the relative expression of *TIM-3* in comparison with the other interventions (Figure 4B). Doxorubicin decreased the level of expression of this gene in 4T1 tumors up to 93.94% compared with placebo. Other chemotherapeutic interventions did not show any statistically significant change in the level of expression of *TIM-3*.

Although doxorubicin showed a trend to decline the level of expression of *CTLA-4* in 4T1 tumor tissue, no statistically significant alterations were evident among groups (Figure 4 C).

**Table 1:** Primer sets for amplification of PD-1, TIM-3, CTLA-4, and β-actin genes

**Gene Name**	**Sequences (5' -> 3')**	**Purification**	**References**
*β-actin-F*	AGAGGGAAATCGTGCGTGAC	HPLC	(51)
*β-actin-R*	CAATAGTGATGACCTGGCCGT	HPLC	
*PD-1-F*	TGCTCAACAAGTATGTCAGAGG	HPLC	(52)
*PD-1-R*	ACACTAGGGACAGGTGCTGC	HPLC	
*Tim-3-F*	CCACGGAGAGAAATGGTTC	HPLC	(52)
*Tim-3-R*	CATCAGCCCATGTGGAAAT	HPLC	
*CTLA4-F*	GCT TCC TAG ATT ACC CCT TCT GC	HPLC	(53)
*CTLA4-R*	CGG GCA TGG TTC TGG ATCA	HPLC	

## Discussion

The goal of this investigation was to determine the effects of three neoadjuvant chemotherapy regimens, including doxorubicin, paclitaxel, and paclitaxel/carboplatin on TILs and immune checkpoints in BC at the preclinical level. Since doxorubicin and paclitaxel are considered the mainstay of NAC regimens in the treatment of triple-negative BC (22), we included them in our experiment. Furthermore, the rationale behind assessment of carboplatin was based on the evidence that the addition of this agent to paclitaxel correlated with better clinical responses compared with standard regimens (23). To avoid the confounding lymphotoxic effects of the administered chemotherapy agents on infiltrating lymphocytes, we assessed the short-term outcome measures instead of long-term effects. 

Our results show that doxorubicin enhances the infiltration of CD3+ and CD8+ subpopulations into the murine mammary gland malignancy within 48 hr.

Although chemotherapeutic agents are endowed with immunosuppressive properties, some preclinical and clinical evidence suggest that these drugs somewhat put on their anti-neoplastic effects via stimulation of the antitumor immunity. Indeed, there are few studies on the impact of NAC on the microenvironment of BC (24). It is essential to mention that the existence of TILs in the residual tumor subsequent to NAC could be connected with a favorable prognosis in the triple-negative subtype of BC (25). After neoadjuvant chemotherapy with doxorubicin-containing regimens, BC residual specimens appeared highly infiltrated by T cells (26).Doxorubicin reportedly increases the infiltration of antigen-specific CD8^+^ lymphocytes in murine mammary gland adenocarcinomas and fibrosarcomas. In this regard, IL-17-producing γδ T cells have been suggested to be required for this event (27). 

An increase in the infiltration of CD8^+^ T lymphocytes in post-treatment tumors can be partly attributed to the release of chemokine interferon-γ inducible protein ten kDa (CXCL10) by doxorubicin-exposed neoplastic cells. Doxorubicin enhances the prompt release of type I interferons in neoplastic cells, which in turn stimulates the release of CXCL10 via autocrine and paracrine mechanisms. Consequently, CXCL10 performs homing functions to chemoattract CD8^+ ^lymphocytes into the tumor bed (28). 

What is more, doxorubicin reportedly up-regulates the natural killer group 2 D receptor (NKG2D) expression exclusively in CD8^+^ T lymphocytes. It has been shown that NKG2D expression is correlated with the accumulation of CD8^+^ lymphocytes in the murine mammary gland tumor (29). 

Increased infiltration of CD8^+^ lymphocytes after neoadjuvant chemotherapy can also be attributed to the induction of immunogenic cell death by doxorubicin in which malignant cells present endoplasmic reticulum chaperone protein calreticulin on the outer leaflet of cell membrane before death and release ATP during apoptosis or non-histone chromatin-binding nuclear protein high mobility group box 1 (HMGB1) upon secondary necrosis. These danger signals lead to infiltration of dendritic cells into the tumor microenvironment and their maturation, which in turn result in priming of CD8^+^ cytotoxic T lymphocytes (30–32).

Our results did not show any significant change in the infiltration of FoxP3^+^ lymphocytes after neoadjuvant chemotherapy in the animal model of triple-negative BC. However, a trend towards decreasing the ratio of intratumoral FoxP3^+^ cells to CD3^+^ lymphocytes was observed in doxorubicin-treated tumor-bearing mice. In support of this possibility, a recent report indicated that this proportion decreases in some patients with triple-negative BC after neoadjuvant chemotherapy comprising anthracyclines and taxanes (33). This alteration has been reportedly linked with favorable prognosis (8, 33). The reason for the non-significant result of our study in this ratio can be due to insufficient sample size or insufficient post-treatment time to detect a meaningful difference. 

This study highlights that doxorubicin down-regulates the expression of immune checkpoints, including *PD-1* and *TIM-3* in the tumor tissue. These findings have not been addressed in previous studies, and anti-immunosuppressive properties of doxorubicin have been mostly contributed to selective elimination of myeloid-derived suppressor cells by this agent (34). On interacting with their relevant ligands, PD-1 and TIM-3 suppress effector T lymphocytes (35). PD-1 suppresses the signaling pathway of T cell receptor and reduces proliferation and cytokine release in T lymphocytes. PD-1 is responsible for intratumoral T_Reg_ cell-mediated immune suppression. TIM-3 represents prominent importance in mediating T lymphocyte exhaustion. Intratumoral TIM-3^+^ lymphocytes also express PD-1 and have impaired function. The high number of TIM-3^+^ lymphocytes has been associated with poor survival in some cancers (6). According to the results of this work, it seems that doxorubicin can inhibit tumor immunosuppression through suppression of *PD-1* and *TIM-3*. In murine models, simultaneous blockade of PD-1 and TIM-3 pathways with targeted agents led to substantial antitumor immune responses (36).

Another hypothesis generated in our study is that carboplatin-containing regimen suppresses the PD-1 gene in the syngeneic model of triple-negative BC. Such an effect was not evident in the group treated only with paclitaxel. Therefore, down-regulation of PD-1 could be attributed to carboplatin in the paclitaxel/carboplatin arm of the experiment. Neither paclitaxel alone nor its combination with carboplatin led to significant alterations in other assessed immune checkpoints or the amount of infiltration of lymphocytes. Review of the literature reveals lack of consensus on the effects of paclitaxel on antitumor immunity. The results of a small prospective study showed that neoadjuvant treatment of breast cancer patients with four cycles of paclitaxel led to induction of TILs (37). Another study indicated the depletion of CD3^+^, CD4^+,^ and CD8^+^ immunophenotypes of lymphocytes after administering taxanes in patients with solid tumors (38). According to the results of an *in vitro* study, after 24-hours of incubation, paclitaxel not only selectively reduced the number of T_Reg_ lymphocytes in the peripheral blood mononuclear cells but also suppressed the inhibitory function of these regulatory cells (39). In the literature search, no study was found to examine the effect of paclitaxel on immune checkpoints. Overall, it can be concluded that some evidence is in favor of boosting antitumor immunity by paclitaxel, and some are against it. To some extent, the underlying cause of these differences could be attributed to the different dosages and schedules of treatment in various studies. We can find the confirmation of this reasoning in studies that assessed the effect of carboplatin on antitumor immunity in different dosages. According to the results of a recent study, carboplatin administration at low dosages led to an increase in CD8^+ ^TILs in lung cancer via inducing DNA breakage and activation of the STING signaling pathway, while high-dosages did not exert such effect. Furthermore, it has been claimed that low-dose carboplatin elevates the expression of programmed death-ligand 1 (PD-L1) in the tumor (40). However, high-dose carboplatin reportedly down-regulated PD-L2 expression in the tumor (41). The dosage of chemotherapeutic agents we used in our experiment was equivalent to high-dose of clinical practice. This dosage seemed to suppress the expression of PD-1 in the paclitaxel/carboplatin group. This result is inconsistent with a recent study that indicated no alteration in PD-1 expression after chemotherapy with paclitaxel/carboplatin in lung cancer patients (42)parent-offspring, friendship. 

Overall, our research generated some novel hypotheses about the effects of main NAC regimens on the down-regulation of immune checkpoint receptors, including *PD-1 *and *TIM-3* in the triple-negative BC. In contrast, literature search reveals that up-regulation of immune checkpoint ligands such as *PD-L1* after chemotherapy has been the focus of publications (43).*PD-L1*, as a ligand of *PD-1* receptor, plays a pivotal role in the suppression of T cell-mediated immune responses. In tumor tissue, this ligand expresses at the surface of neoplastic cells. *PD-L1* and *PD-1* interaction lead to the escape of neoplastic cells from antitumor immunity (44). According to the results of a study, platinum derivatives increased the expression level of *PD-L1 *on both head and neck squamous cell carcinoma cell lines and patient tumors (45). Similar findings have been observed after administration of gemcitabine and 5-fluorouracil (46, 47). Based on such observations, some researchers have proposed that co-administration of immune checkpoint blockers with chemotherapeutic agents like platinum derivatives might be more advantageous for patients (48, 49). However, the generated hypothesis of our work about the probability of down-regulation of immune checkpoint receptors by doxorubicin and paclitaxel could raise new questions about the efficacy of co-administration of these agents with immunotherapy drugs. 

The assessment of NAC regimens’ efficacy on triple-negative BC was out of the scope of our aim. Since the objective of this work was to assess the short-term alterations in TILs and immune checkpoints after NAC, we excluded tumor size changes and kinetics of tumor growth from the outcome measures. Indeed tumor size alterations cannot be discerned within 48 hr after treatment.

Another limitation of the present study can be attributed to the interpretation of results based on one cell line-derived triple-negative syngeneic tumor. Utilizing further cell lines is needed for generalizing these results with great confidence. Another limitation of this work was that the immunophenotype of immune-checkpoint expressing lymphocytes was not assessed. Evaluation of PD-1, TIM-3, and CTLA-4 expressing cells by flowcytometry can elucidate this issue in the future studies. 

It is worth noting the nature of the present study is exploratory, not confirmatory. The main goal of exploratory studies is to generate hypotheses. The findings of this work generated new hypotheses about the down-regulation of some immune checkpoints by chemotherapeutic agents like doxorubicin and carboplatin-containing regimens. However, these exploratory hypotheses can only be tentative and need to be tested in confirmatory study settings with adequate sample sizes, more rigorous methodology, and specific assays (50).

## Conclusion

Overall, our findings indicate that NAC with doxorubicin potentiates antitumor immunity not merely by recruitment of TILs, but via down-regulation of PD-1 and TIM-3 checkpoints. Carboplatin-containing NAC may suppress *PD-1* as well. However, these results raise new questions about the efficacy of immunotherapy with PD-1 blocking agents subsequent to these NAC regimens. Further research on host, tumor, and microenvironmental parameters is needed to elucidate the mechanism of down-regulation of these checkpoints and their effects on choosing a combination of chemotherapy, targeted agents, and immunotherapy drugs in the neoadjuvant setting.
